# Hot Hole Enhanced Synergistic Catalytic Oxidation on Pt‐Cu Alloy Clusters

**DOI:** 10.1002/advs.201600448

**Published:** 2017-02-23

**Authors:** Lanchun Zhang, Chuancheng Jia, Shuren He, Youtao Zhu, Yana Wang, Zhenhuan Zhao, Xiaochun Gao, Xiaomei Zhang, Yuanhua Sang, Dongju Zhang, Xiaohong Xu, Hong Liu

**Affiliations:** ^1^ Key Laboratory of Colloid and Interface ChemistryMinistry of EducationSchool of Chemistry and Chemical EngineeringShandong University27 Shandanan RoadJinan250100China; ^2^ Department of Chemistry and BiochemistryUniversity of CaliforniaLos AngelesCA90095USA; ^3^ State Key Laboratory of Crystal MaterialsShandong University27 Shandanan RoadJinan250100China

**Keywords:** alloy cluster, catalytic oxidation, hot carrier, interband excitation, photocatalysis

## Abstract

**Hot holes in Pt‐Cu alloy clusters** can act as catalyst to accelerate the intrinsic aerobic oxidation reactions. It is described that under visible light irradiation the synergistic alcohol catalytic oxidation on Pt‐Cu alloy clusters (≈1.1 nm)/TiO_2_ nanobelts could be significant promoted by interband‐excitation‐generated long‐lifetime hot holes in the clusters.

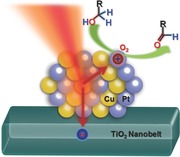

For supported noble metal nanoparticle catalysts in conventional heterogeneous catalysis, it is desirable to reduce the metal particle size to increase the surface atoms of metal, which improves the activity and cost economy of catalysts. However, extremely small sized nanoparticles, such as the metal clusters with the size of ≈1 nm, are very unstable at high reaction temperature. Luckily, photoassisted catalysis over metal nanostructure provides an approach to allow for chemical transformations being performed at room temperature with visible light irradiation.^1^, ^2^, ^3^, ^4^

The photoassisted catalysis of nanostructured metals starts at the generation of hot carriers within photoexcited metal nanostructures, either by direct photoexcitation or plasmon decay.^5^ Plasmon decay is the most excited and intensively studied hot carrier generation mechanism. In this process, the large absorption cross sections of plasmonic nanostructures give strong optical absorption, as well as greatly enhanced photoexcitation and hot‐carrier generation. So in recent years, tremendous research efforts have focused on the plasmonic hot electrons driven photoassisted catalysis of typical plasmonic metals (Au and Ag) with the feature sizes larger than 2 nm.^2^, ^3^, ^4^ In contrast, only little attention has been paid to the photochemical process induced by the direct photoexcitation, which may be due to the relatively inefficient optical absorption and hot‐carrier generation. However, for metal cluster smaller than 2 nm, especially the nonplasmonic metal ones (such as Pt) that are the most widely used and efficient catalysts for organic transformations, direct photoexcitation will dominate the generation of visible light induced hot carries. The theoretical investigations on hot‐carrier generation have described that the interband transition (d to s states) can generate high energy hot d‐band holes and electrons with only modest energies.^6^, ^7^, ^8^ Recently, the visible light‐excited Pt nanoparticles with the size greater than 2 nm have been reported to be high reactive for various redox reactions at room temperature.^9^, ^10^, ^11^, ^12^ However, the role of photogenerated hot carriers in Pt nanostructures for enhancing catalysis still remains to be further identified.

For the direct photoexcitation of Pt metal clusters, energetic hot d holes with strong oxidizing power can be expected by interband transition. However, the energetic hot d holes have too short mean free paths (<1 nm) to be efficiently used in photocatalysis for large‐size metal nanostructures.^6^ Evidently, decreasing the size of metal nanostructures, in particular to the size scale comparable with the mean free paths of ≈1 nm, the energetic d holes will become more available for catalyzing chemical transformation by trapping the electrons at the occupied molecular orbitals of adsorbates. Therefore, it is of crucial importance to prepare ultrasmall Pt based nanostructures for realizing highly efficient photoassisted catalysis.

In conventional heterogeneous catalysis, enhanced activities are commonly obtained by mixing two different metals to form alloy nanostructure, because alloying can create a unique electron structure that is different from either of the single components.^13^, ^14^ What is more, such alloying often results in the decrease of metal nanoparticles size,^15^, ^16^ which is usually advantageous for thermocatalysis, and should also favor photoassisted catalysis driven by hot holes. Meanwhile, a significant promotion for the generation and/or separation of photogenerated carriers can also be expected due to the formation of novel energy structure in alloy structure.^9^, ^15^ When the metal clusters are further dispersed on semiconductor supports with matched energy levels (such as TiO_2_), the efficient transfer of hot electrons at heterointerface will increase the lifetime of short‐lived holes, and make metal clusters positive charging and more reactive for various oxidation reactions.

In this work, we design and synthesize PtCu/TiO_2_‐NB hybrid nanostructures to explore the hot holes driving photoassisted catalysis process. The PtCu/TiO_2_‐NB nanostructures consist of ultrasmall Pt‐Cu alloy clusters (≈1.1 nm) highly dispersed on heterostructured TiO_2_(B)/anastase nanobelts (NBs) that have smooth surface for uniformly dispersing metal clusters^16^, ^17^ and remarkable axial direction carriers transport properties for effectively transferring the electrons.^18^, ^19^ The aerobic oxidation of alcohols, an important transformation process for the production of fine chemicals,^20^ was chosen to evaluate the hot hole‐driven photoassisted catalysis of PtCu/TiO_2_‐NB. This is because in the proposed mechanism,^20^, ^21^ the key steps involve the adsorption of alcohols and the subsequent cleavage of O—H and C—H bonds to form metal hydride (M‐H), for which the positively charged metal particles should be favorable and can activate the overall reaction.

For the fabrication of PtCu/TiO_2_‐NB nanostructures, TiO_2_(B)/anatase heterostructured NBs with a width of 50–200 nm and length of up to dozens of micrometers (Figures S1 and S2, Supporting Information) were first synthesized via a typical hydrothermal process.^22^, ^23^ Based on these TiO_2_ NBs, PtCu/TiO_2_‐NB nanostructures with different Pt/Cu ratios were synthesized by a facile deposition–precipitation method (Scheme S1, Supporting Information). Inductively coupled plasma (ICP) analysis indicates that the total metal contents in all obtained nanomaterials are approximate to a nominal value of 0.5 wt% and the Pt/Cu ratios are also close to the nominal values (Table S1, Supporting Information). Transmission electron microscopy (TEM) images show large amounts of ultrasmall metal clusters with narrow size distribution are evenly dispersed over the TiO_2_ NBs surface (**Figure** **1**a and Figures S3 and S4, Supporting Information). The average diameters of Pt‐Cu clusters with different Pt/Cu ratios are around 1.1 nm, while the Cu and Pt clusters show an average size of 1.96 and 1.49 nm, respectively. The high‐resolution image (HR‐TEM), taking Pt_1_Cu_1_/TiO_2_‐NB as an example, depicts well‐crystallized clusters with a lattice spacing of ≈1.89 Å that is just between the values of Cu (200) (1.80 Å) and Pt (200) (1.96 Å), indicative of a stable Pt‐Cu alloy structure (Figure 1b). Furthermore, for the X‐ray photoelectron spectroscopy (XPS) analysis, the binding energies of Cu 2p (932.1 and 951.9 eV) (Figure S5b, Supporting Information) and Pt 4f (71.3 and 74.8 eV) (**Figure** **2**b) in Pt_1_Cu_1_/TiO_2_‐NB nanostructure can be respectively assigned to metallic Cu and metallic Pt, and the feature peak corresponding to CuO*_x_* was not detected. These indicate that the oxidation of Cu atoms is effectively suppressed in bimetallic Pt‐Cu alloy clusters, which is distinct different from that of pure Cu clusters (Figure S5a, Supporting Information). Consequently, stable ultrasmall Pt‐Cu alloy clusters were successfully prepared on the surface of TiO_2_ NBs.

**Figure 1:**
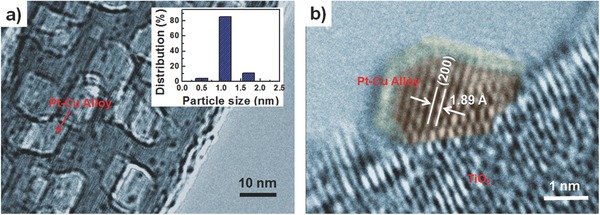
a) Typical TEM image of Pt_1_Cu_1_/TiO_2_‐NB nanostructures. The inset shows corresponding size distributions of Pt‐Cu clusters. b) Typical HR‐TEM image of Pt_1_Cu_1_/TiO_2_‐NB nanostructure.

**Figure 2:**
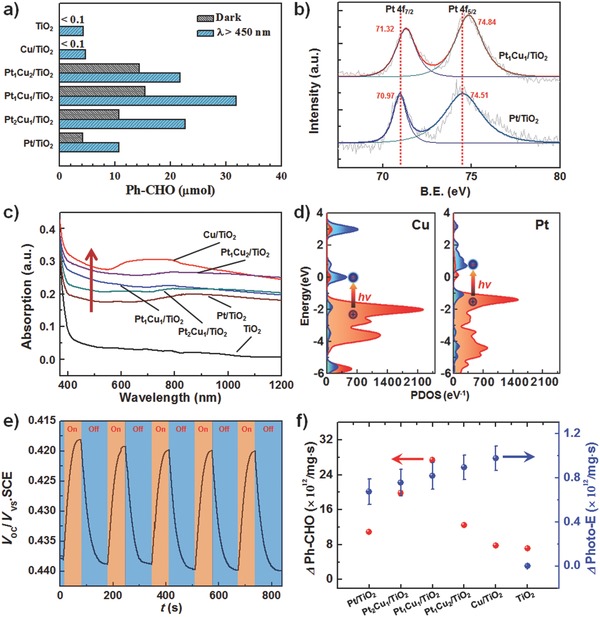
a) Amounts of benzaldehyde formed during benzyl alcohol oxidation in dark (gray) and under visible light (cyan) over bare TiO_2_ NBs and Pt‐Cu/TiO_2_‐NB with different Pt/Cu ratios. b) XPS spectra of Pt 4f in 1.5 nm Pt/TiO_2_‐NB and 1.1 nm Pt_1_Cu_1_/TiO_2_‐NB. c) UV–Vis–NIR diffuse reflectance spectra for different nanostructures. d) Projected density of states (PDOS) spectra for model Cu_13_ (left) and Pt_13_ (right) clusters, where PDOS of d orbits are marked in orange red color and PDOS of sp orbits are marked in ultramarine color. The hot carrier generation processes are also shown out, as electrons near Fermi level are photoexcited from d to sp orbitals. e) Transient open‐circuit voltage (*V*_oc_) rise/decay obtained during alternant excitation/termination of irradiation. f) Compared with benzyl alcohol oxidation in dark, increased number of product molecules (ΔPh‐CHO, red) and corresponding number of photogenerated electrons (Δ*I*_light_, blue) on per unit mass (mg) different nanostructures per second.

With the prepared PtCu/TiO_2_‐NB nanostructures, the intrinsic benzyl alcohol aerobic catalytic oxidation in the dark was first studied. The amounts of benzaldehyde formed after 5 h reaction over 20 mg PtCu/TiO_2_‐NB with different Pt/Cu ratios are shown in Figure 2a (gray) and Table S2 (Supporting Information). Remarkably, benzyl alcohol was converted to benzaldehyde with high selectivity of >99% for all catalysts. All bimetallic PtCu/TiO_2_‐NB catalysts exhibited improved activity compared to Pt/TiO_2_‐NB, which inherently has weak activity for benzyl alcohol oxidation in the dark. In addition, a volcano‐type relationship between catalytic activity and Pt/Cu ratio was observed, and Pt_1_Cu_1_/TiO_2_‐NB showed the highest activity. By contrast, both bare TiO_2_ NB and monometallic Cu/TiO_2_‐NB with CuO*_x_* states exhibited no activity in the dark, indicating that both TiO_2_ NB and CuO*_x_* are not catalytic active for the benzyl alcohol oxidation. Furthermore, when the Cu atoms in PtCu/TiO_2_‐NB were oxidized into CuO*_x_* states by calcining as‐deposited Pt_1_Cu_1_/TiO_2_‐NB sample in oxygen atmosphere, the produced benzaldehyde was greatly decreased from 15.4 to 3.4 µmol for dark reactions (Figure S6, Supporting Information). These results indicate that Pt‐Cu clusters have a synergistic catalytic activity for benzyl alcohol oxidation in dark, where alloying structure of the clusters plays a key role.

In order to understand such synergistic catalysis processes on Pt‐Cu clusters, the energy level structures for Pt atoms in Pt/TiO_2_‐NB and Pt_1_Cu_1_/TiO_2_‐NB were studied by XPS analysis. Positive shift of Pt 4f_7/2_ and 4f_5/2_ peaks in Pt_1_Cu_1_/TiO_2_‐NB with respect to those in Pt/TiO_2_‐NB was observed (Figure 2b), indicating that Pt atoms in alloy clusters are more positively charged.^24^ Through considering both work function of intrinsic metal and small size effect,^25^, ^26^ the work function for origin 1.5 nm Pt cluster is calculated of 6.37 eV, which is 6.13 eV for origin 1.1 nm Pt_1_Cu_1_ cluster (Table S3, Supporting Information). Therefore, the observed positive charge is possibly due to the fact that the superficial Cu atoms in Pt_1_Cu_1_ cluster bind with O_2_ molecules, thus down‐shifting the intrinsic energy level of the clusters.

For Pt‐Cu alloy cluster in dark reaction (**Figure** **3**a), Cu atoms bind with dissolved O_2_ molecules to form activated oxygen on superficial Cu sites.^27^, ^28^ This leads to the partially positive charging of the Pt atoms adjacent to Cu atoms, which expedites the formation of the carbonylic derivative on Pt sites. Moreover, the activated oxygen on Cu sites can directly react with the generated metal‐H intermediate. Thus, synergistic catalytic oxidation with high efficiency is carried out on Pt‐Cu clusters.

**Figure 3:**
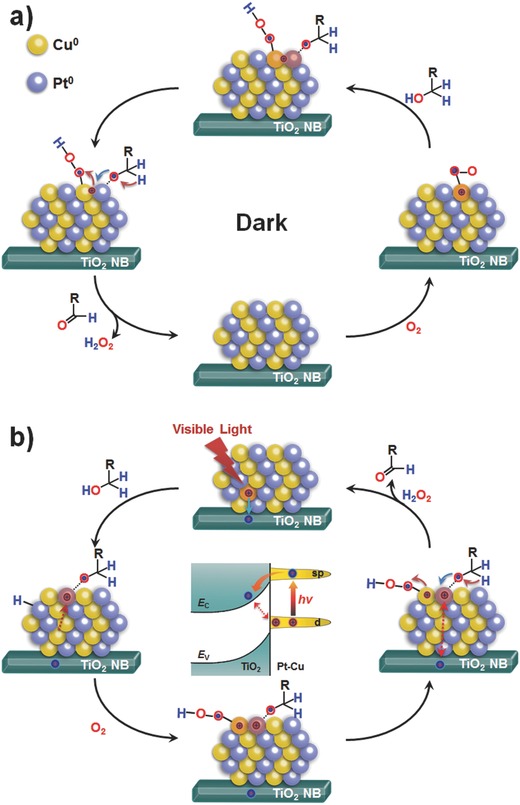
a) Schematic of synergistic catalytic processes for alcohol aerobic oxidation on Pt‐Cu/TiO_2_‐NB in dark. b) Schematic of hot carrier enhanced synergistic catalysis catalytic processes for alcohol aerobic oxidation on Pt‐Cu/TiO_2_‐NB under visible light. The inset at center shows an energy band diagram for hot carrier generation and transfer at the PtCu/TiO_2_‐NB interface.

Under visible light irradiation (Xe lamp, λ > 450 nm), photoassisted catalytic processes were further studied. For TiO_2_ NBs and Cu/TiO_2_‐NB, only a little amount of benzaldehyde was produced under visible light irradiation, which is attributed to the ligand‐to‐metal charge transfer for the benzyl alcohol directly adsorbed on the surface of TiO_2_ NBs.^29^ While, for Pt/TiO_2_‐NB and PtCu/TiO_2_‐NB systems, as shown in Figure 2a (cyan) visible light irradiation remarkably prompted the reaction of benzyl alcohol oxidation, especially for Pt_1_Cu_1_/TiO_2_‐NB with a maximum light‐driven activity enhancement of 16.4 µmol (Figure S7a, Supporting Information).

For the purpose of understanding such photoassisted activity enhancement of PtCu/TiO_2_‐NB, the optical absorption properties of PtCu/TiO_2_‐NB as well as the monometallic counterparts were examined (Figure 2c). In contrast to the bare TiO_2_ NBs that only absorb at wavelength below 400 nm, a broad absorption in visible and near infrared region appears for Pt/TiO_2_‐NB, which is ascribed to interband excitation (5d to 6sp) of Pt atoms.^30^ For PtCu/TiO_2_‐NB, with increasing Cu/Pt ratio, a remarkable increase of absorption in the visible region can be observed. This is due to the plasmon‐induced interband excitation (3d to 4sp) of metallic Cu.^31^ While for Cu/TiO_2_‐NB a quite broad absorption band from 580 to 800 nm occurs, which can be attributed to the existence of CuO*_x_* species.^32^ Furthermore, from projected density of states (PDOS) calculated from model Cu_13_ and Pt_13_ clusters (Figure 2d and Figure S8, Supporting Information), it can be observed that near Fermi level the PDOS of d orbits for Cu is larger than that for Pt. Therefore, the metallic Cu atoms in Pt‐Cu alloy clusters can generate more hot carriers through interband excitation from d to sp orbitals, thus enhancing the photoassisted catalytic oxidation on the alloy clusters.

In addition, with PtCu/TiO_2_‐NB or bare TiO_2_ NBs as working photoanodes, photocurrent and photovoltage measurements were carried out in a three‐electrode system to explore the role of hot carriers in the photoassisted catalysis. All as‐prepared catalysts, except bare TiO_2_, showed significant anodic photoresponse with visible light irradiation. Take Pt_1_Cu_1_/TiO_2_‐NB as an example (Figure 2e), transient open‐circuit voltage (*V*_oc_) rise can be observed during light irradiation, which is ascribed to hot electrons injection from Pt_1_Cu_1_ clusters to TiO_2_ NBs with hot holes left in the clusters. After light irradiation, transient *V*_oc_ decay with an average rate parameter of 23.21 ± 0.84 s can be obtained (Table S4, Supporting Information). This indicates that Schottky barrier between clusters and TiO_2_ NBs combined with the good electrons transport properties of heterostructured TiO_2_ NBs can realize efficient separation of photogenerated electron–hole pairs with generating long‐lived hot holes in clusters.^33^ For photocurrent response, with increasing Cu/Pt ratio enhanced photocurrent can be observed (Figure S9, Supporting Information). Biggest photocurrent response was obtained for Cu/TiO_2_‐NB with CuO*_x_* states, which is consistent with its optical absorption characteristics (Figure 2c), but has no contribution to the catalytic reactions (Figure 2a). This unambiguously indicates that the light‐promoted aerobic oxidation reactions on PtCu/TiO_2_‐NB are a metal cluster dominated photocatalytic process and that the breakage of O—H and C—H bonds of alcohol upon adsorption on metallic active sites is the key step to achieve the conversion of alcohol to carbonylic compounds. From further comparing the increased number of product molecules under photoassisted catalysis and corresponding number of photogenerated electrons on per unit mass (mg) catalysts per second (Figure 2f), a volcano‐type relationship between generated molecules/electrons ratios and Cu/Pt ratios was also observed (Figure S7b, Supporting Information), and a maximum ratio of 33.5 was obtained for Pt_1_Cu_1_/TiO_2_‐NB. Hence, it can be reckoned that one hot hole in Pt‐Cu clusters can bring out the generation of more than one order of magnitude additional product molecules.

Based on the above results and analysis, the mechanism for photoassisted catalytic oxidation on PtCu/TiO_2_‐NB is possible as follows (Figure 3b). Under visible light irradiation, the interband transition from d to sp orbitals occurs in Pt‐Cu clusters with generation of hot electron–hole pairs, especially for the contained metallic Cu atoms with larger PDOS of 3d orbitals. The hot electrons tunnel across the Schottky barrier and inject into the conduction band of TiO_2_ NBs, and the long‐lived hot d holes with high energies are left on the clusters. The left hot holes, positively charging the clusters, can be used as catalysts to accelerate the intrinsic synergistic catalytic process on Pt‐Cu clusters during the aerobic oxidation reactions.

To verify the proposed hot hole enhanced synergistic catalytic oxidation mechanism, Pt_1_Cu_1_/TiO_2_‐NB with different cluster sizes were further studied. Through varying annealing time from 2 to 4 and 6 h, Pt‐Cu clusters with average sizes of 1.3 and 1.5 nm were successfully prepared, which were confirmed by TEM characterizations (**Figure** **4**a). The XPS spectra showed that both Pt 4f and Cu 2p peaks shift to the higher binding energies with decreasing cluster sizes (Figure 4b), confirming that decreasing the cluster size makes Pt‐Cu clusters more positively charged, which is consistent with the small size effects of metal clusters.^24^ As shown in Figure 4c, the size of Pt‐Cu clusters does have a strong impact on the catalytic performance of Pt_1_Cu_1_/TiO_2_‐NB for benzyl alcohol oxidation with or without light irradiation. When the average size of clusters increased from 1.1 to 1.3 and 1.5 nm, the formed amount of benzaldehyde decreased from 15.4 to 10.3 and 9.4 µmol for dark reactions, and the light‐driven increment of benzaldehyde produced significantly decreases from 16.4 to 10.1 and 6.7 µmol. Furthermore, the photoelectrochemical (PEC) measurements also showed a decrease in photocurrent response with increasing cluster sizes. As shown in the inset of Figure 4c and Figure S10 (Supporting Information), the average photocurrent density measured for Pt_1_Cu_1_/TiO_2_‐NB is decreased from 25.7 nA cm^−2^ for 1.1 nm clusters to 20.5 and 18.2 nA cm^−2^ for 1.3 and 1.5 nm clusters, respectively. In addition, similar size effects also took place in the Pt/TiO_2_‐NB system (Figure S11, Supporting Information). Such strong size effects for photoassisted catalytic and photoelectrochemical performances of metal cluster smaller than 2 nm may be largely attributed to the shorter carrier travel length of hot carriers, which can greatly reduce the hot carriers recombination and scattering during the transport in the clusters, thus realizing more efficient injection of hot electrons into TiO_2_ and thereby more hot holes are available for driving the aerobic oxidation.

**Figure 4:**
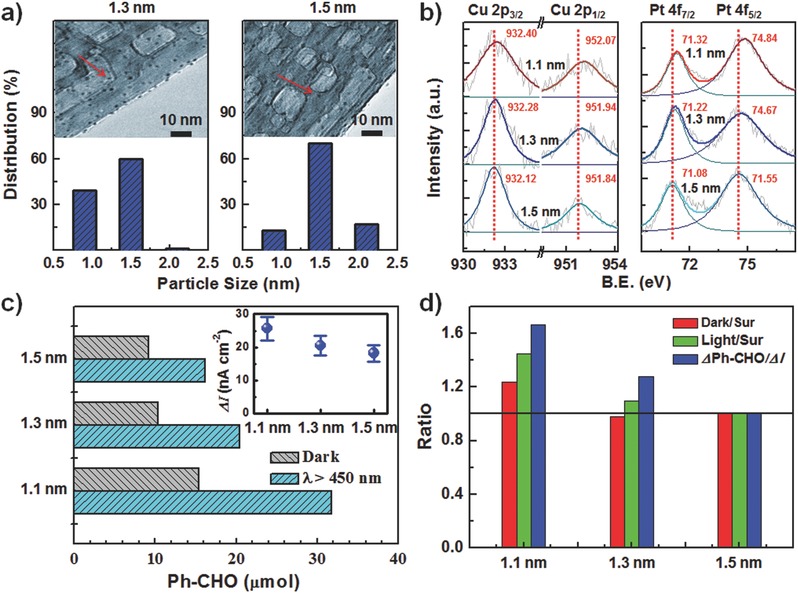
a) Cluster size distributions and corresponding typical TEM images for Pt_1_Cu_1_/TiO_2_‐NB with Pt‐Cu cluster sizes of 1.3 nm (left) and 1.5 nm (right). b) XPS spectra of Cu 2p (left) and Pt 4f (right) for Pt_1_Cu_1_/TiO_2_‐NB with cluster sizes of 1.10, 1.3, and 1.5 nm. c) Amounts of benzaldehyde formed during benzyl alcohol oxidation in dark (gray) and under visible light (cyan) over Pt_1_Cu_1_/TiO_2_‐NB with different cluster sizes. The inset is corresponding photocurrent response under the same visible light. d) Catalytic activity ratio between Pt‐Cu clusters with different cluster sizes and 1.5 nm clusters. Dates in red are for dark reactions and dates in green are for light‐assisted reactions, which are normalized to surface area of clusters; dates in blue are for increment of light‐assisted reactions, which are normalized to photocurrent.

For exploring intrinsic catalytic processes on the surface of Pt‐Cu clusters, catalytic activities are normalized to unit surface area of clusters. For the dark reactions, from the catalytic activity ratios between Pt‐Cu clusters with different cluster sizes and 1.5 nm Pt‐Cu clusters (red in Figure 4d), distinct enhanced superficial catalytic activity (≈1.25 times) for 1.1 nm Pt‐Cu clusters can be observed. This result demonstrates that the more positively charged Pt‐Cu clusters are more catalytic active for alcohols aerobic oxidation. For the photoassisted reactions, with decreasing size of the clusters from 1.5 to 1.3 and 1.1 nm, the superficial catalytic activities are increased to 1.10 and 1.44 times, respectively. More significantly, when the increment catalytic activities under light irradiation are normalized to unit photocurrent, the increment catalytic activities are increased to 1.30 and 1.66 times for 1.3 and 1.1 nm clusters, respectively. These results further prove that the hot holes in Pt‐Cu clusters can accelerate intrinsic catalytic oxidation reactions on the surface of clusters, especially for the ultrasmall clusters (1.1 nm) that have high intrinsic catalytic activities and efficient hot‐carriers generation.

In summary, PtCu/TiO_2_‐NB nanomaterials composed of stable ultrasmall Pt‐Cu alloy clusters (≈1.1 nm) highly dispersed on TiO_2_ NBs were synthesized by a facile deposition–precipitation method. In the dark, synergistic catalytic oxidation of benzyl alcohol takes place on the Pt‐Cu clusters with high efficiency. When the Pt‐Cu alloy clusters were illuminated by visible light, interband transitions, especially for the metallic Cu atoms in the clusters, are effectively excited for generating hot electron–hole pairs. The hot electron–hole pairs are further separated by Schottky barrier at cluster/TiO_2_ interfaces with producing long‐life hot holes in clusters. Such hot holes can efficiently accelerate the intrinsic synergistic catalytic oxidation reactions on Pt‐Cu clusters, especially for clusters with smaller sizes, thus realizing high visible light‐promoted activity for PtCu/TiO_2_‐NB catalysts.

## Experimental Section

Experimental details are included in the Supporting Information.

## Supporting information

As a service to our authors and readers, this journal provides supporting information supplied by the authors. Such materials are peer reviewed and may be re‐organized for online delivery, but are not copy‐edited or typeset. Technical support issues arising from supporting information (other than missing files) should be addressed to the authors.

Supplementary
