# Supplementary material for: Hot Hole Enhanced Synergistic Catalytic Oxidation on Pt‐Cu Alloy Clusters
Source: Adv Sci (Weinh). 2017 Feb 23;4(6):1600448. doi: 10.1002/advs.201600448 (PMC5473327; doi:10.1002/advs.201600448)
Supplement: Supplementary file 1 — Supplementary [file ADVS-4-na-s001.pdf]

Copyright WILEY-VCH Verlag GmbH & Co. KGaA, 69469 Weinheim, Germany, 2017.

ADVANCED SCIENCE
Open Access

Supporting Information

for *Adv. Sci.,* DOI: 10.1002/advs.201600448

Hot Hole Enhanced Synergistic Catalytic Oxidation on Pt-Cu Alloy Clusters

Lanchun Zhang, Chuancheng Jia, Shuren He, Youtao Zhu, Yana Wang, Zhenhuan Zhao, Xiaochun Gao, Xiaomei Zhang, Yuanhua Sang, Dongju Zhang, Xiaohong Xu,* and Hong Liu**

Copyright WILEY-VCH Verlag GmbH & Co. KGaA, 69469 Weinheim, Germany, 2016.

Supporting Information

Hot Hole Enhanced Synergistic Catalytic Oxidation on Pt-Cu Alloy Clusters

Lanchun Zhang, Chuancheng Jia, Shuren He, Youtao Zhu, Yana Wang, Zhenhuan Zhao, Xiaochun Gao, Xiaomei Zhang, Yuanhua Sang, Dongju Zhang, Xiaohong Xu*, and Hong Liu**

L. Zhang, S. He, Y. Zhu, X. Gao, Prof. X. Zhang, Prof. D. Zhang, Prof. X. Xu
Key Laboratory of Colloid and Interface Chemistry, Ministry of Education, School of Chemistry and Chemical Engineering, Shandong University, 27 Shandanan Road, Jinan 250100, China
E-mail: xhxu@sdu.edu.cn
Dr. C. Jia
Department of Chemistry and Biochemistry, University of California, Los Angeles, California 90095, USA
E-mail: jiacc@chem.ucla.edu
Y. Wang, Z. Zhao, Y. Sang, Prof. H. Liu
State Key Laboratory of Crystal Materials, Shandong University, 27 Shandanan Road, Jinan 250100, China
E-mail: hongliu@sdu.edu.cn

This file includes:

Materials and Methods
Figure S1–S11
Table S1–S4
Reference list

1. Details of Experiments

1.1 Materials

Commercial P25 (Evonik, 50 m^2 g^{-1}, 80% anatase, 20% rutile) powder was hydrothermal treated in concentrated NaOH aqueous solution to synthesize the TiO_2 nanobelts.[1,2] The calcination temperature of $H_2Ti_3O_7$ nanobelts is 873 K to obtain TiO_2 nanobelts (NBs) with a composition of 40% TiO_2(B) and 60% anatase. H_2PtCl_6 and $Cu(CH_3COO)_2$ (Acros Chemicals) were used as platinum and copper precursors without further treatment. Ultrapure water was used throughout our experiments.

1.2 Synthesis of M/TiO_2-NB nanostructure (M = Pt, Cu, Pt-Cu, Pt-CuO_x)

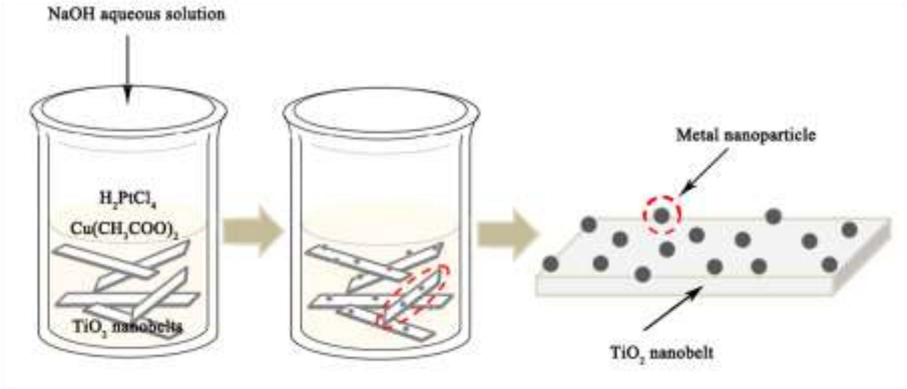

Scheme S1. Schematic illustration for the synthesis of PtCu/TiO_2-NB nanostructures.

PtCu/TiO_2-NB: The PtCu/TiO_2-NB nanomaterials were prepared by the deposition-precipitation method (Scheme 1). TiO_2 NBs (0.1 g) were dispersed evenly in 50 mL aqueous solution comprising H_2PtCl_6 and $Cu(CH_3COO)_2$ with a controlled Pt/Cu molar ratio, and the suspension was vigorously magnetic stirred for half an hour to reach adsorption equilibrium. Subsequently, the pH of the precursor solution was adjusted to 8 with NaOH aqueous solution. Thereafter, the suspension was thermostatically held at 353 K and kept for 4 h under vigorous magnetic stirring. The whole reaction was carried out in the absence of light. The resulting product was filtrated and washed for several times, then dried in air at 353 K for 12 h, and annealed at 673 K for 2 h with a 5 K min^{-1} heating rate under H_2 flow.

Cu(Pt)/TiO_2-NB: The monometallic Cu/TiO_2-NB and Pt/TiO_2-NB nanomaterials were synthesized by the same deposition-precipitation method as that for PtCu/TiO_2-NB.

Pt-CuO_x/TiO_2-NB: The Pt-CuO_x/TiO_2-NB nanomaterials was synthesized by the same deposition-precipitation method as that for PtCu /TiO_2-NB, only the sample was obtained by calcinating at 673K for 1 h under H_2 flow and 1 h under O_2 flow with a 5 K min^{-1} heating rate.

1.3 Catalysts characterization

The metal loading and the Pt/Cu molar ratio in the as-prepared samples were analyzed by an inductively coupled plasma spectrometer (ICP-AES) on an IRIS Intrepid II XSP instrument (Thermo Electron Corporation). X-ray diffraction (XRD) analysis was conducted on a German Bruke D8 Advance powder X-ray diffractometer with Cu-Kα (λ = 0.15406 nm). Transmission electron microscopy (TEM) and high resolution transmission electron microscope (HR-TEM) images were obtained with a JOEL JEM 2100 microscope. X-ray photoelectron spectroscopy (XPS) data was acquired on a Thermo ESCALAB 250 X-ray photoelectron spectrometer and the binding energies were determined utilizing C1s spectrum as reference at 284.6 eV. The UV-Vis-NIR absorption spectra were recorded on a UV-Vis-NIR spectrophotometer (Varian Cary 5000) with dual beam capability in the range of 200-2600 nm.

1.4 Catalytic reaction tests

The photocatalytic activities of the M/TiO_2-NB nanostructures were estimated via the aerobic oxidation of benzyl alcohol under visible light irradiation. The catalyst (20 mg) was dispersed evenly by ultra-sonication in a Pyrex glass tube (15 mm in diameter with a capacity of 20 mL) containing 5 mL toluene and 40 μmol benzyl alcohol. The tube was sealed with a rubber septum cap at once after purging the suspension with O_2 for 5 min. The irradiation was carried out under vigorous magnetic stirring for 5 h using a 500 W Xenon lamp with a 450 nm cut-off filter (160 mW cm^{-2}) as the light source. The temperature of the system was controlled by a water bath (303 ± 0.5 K) running through the outer casing of the Pyrex glass tube to avoid light induced heating. The control experiment was carried out under the same condition in the dark. After the reaction, the suspension was separated by centrifugation, and the supernatant was analysed with Shimadzu Type GC-14C equipped with a flame ionization detector, using a SGE-30QC2/AC5 capillary column and N_2 as carrier gas.

1.5 Photocurrent and photovoltage measurements

The photoelectrochemical measurements were performed using a classical three-electrode cell, with Pt counter electrode and a saturated calomel reference electrode (SCE). A ITO-glass coated with M/TiO_2-NB nanostructures served as the as the photoanode (working

electrode). 20 mg catalyst was mixed Nafion/ethanol (v/v=1:9) to form a slurry which was coated directly onto an ITO-glass (2 cm × 2 cm) usingg a spin-coater (chemat technology, Kw-4A). The coated ITO-glass was dried at room temperature in vacuum and then calcined at 473 K for 4 h under N_2 flow. The photocurrent and photovoltage responses were recorded at zero bias voltage on a CHR650D electrochemical workstation (the current density was normalized by the geometric surface area of the electrode) using 0.2 M Na_2SO_4 as the electrolyte solution. The working electrode was alternately irradiated by a 500 W Xenon lamp with a 450 nm cut-off filter (160 mW cm^{-2}).

1.6 Computational models and methods

The cuboctahedral Pt_{13} (O_h symmetry) and icosahedral Cu_{13} (I_h symmetry) cluster computed in this study, are considered as the lowest-energy isomers.[3–6] All the calculations were carried out using the Dmol3 code[7,8] in Materials Studio(Vesion 5.0) (Accelrys Inc, USA). Geometries of the clusters were optimized using the generalized gradient approximation (GGA) with the Perdew-Wang91 (PW91) correlation functional at the Double Numerical plus d-functions (DND) basis set level.[9,10]

First, the two clusters were fully optimized with respective symmetry, and vibrational frequency calculations were also performed for the two lowest-energy clusters to ensure that they were true minima on the potential energy surface. Then, the optimized clusters were placed in a large cubic unit cell (20×20×20 Å) with periodic boundary conditions. The Brillouin zone was sampled by 1×1×1 k-points. The convergence criterion for force is set to be within 1×10^{-5}eV.

2. Figure S1–S11

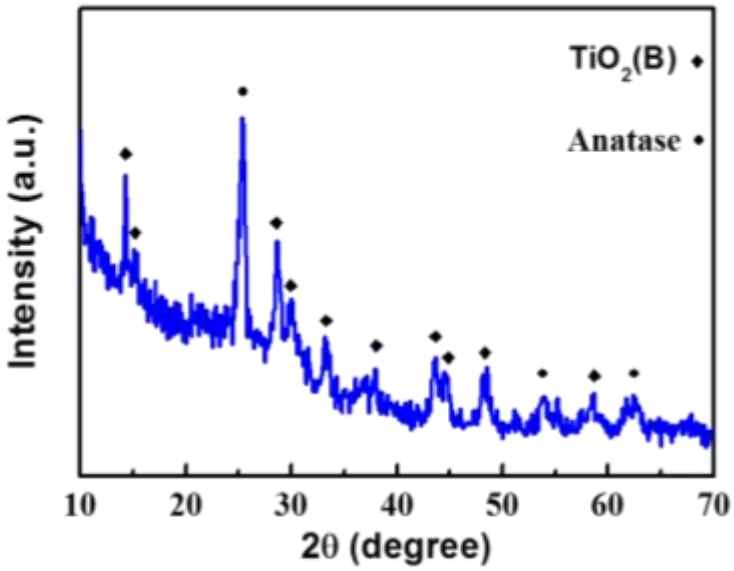

Figure S1. XRD patterns of TiO_2 NBs, which are composed of TiO_2(B) (monoclinic, space group *C*2/*m*), which is often formed as a metastable interphase between $H_2Ti_3O_7$ and anatase, and anatase (tetragonal, space group *I*4_1/*amd*). [11]

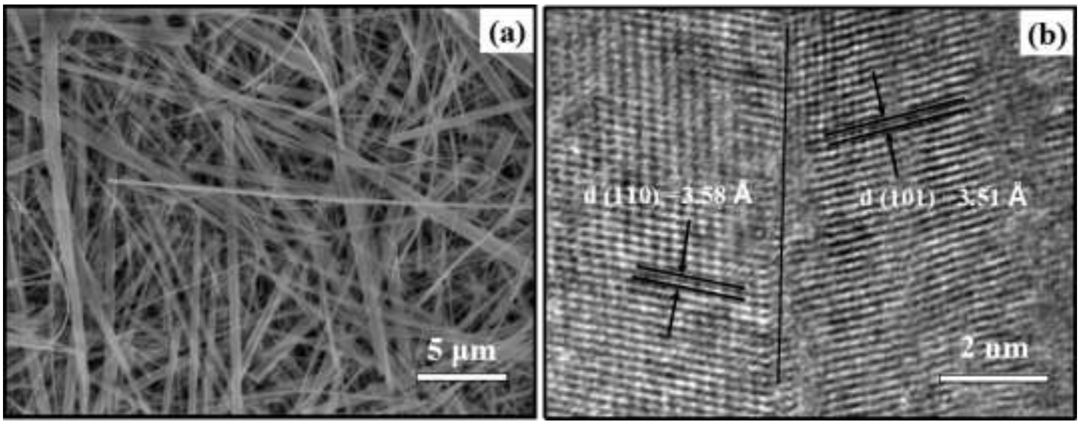

Figure S2. (a) Typical SEM image and (b) HR-TEM image of TiO_2 NBs. The HR-TEM image displays that two clear lattice fringes continuously spaced across the surface of TiO_2 NBs. The interplanar distances of 3.51 Å and 3.58 Å can be respectively indexed to (101) plane of anatase and (110) plane of TiO_2(B), and the interface of the two phases is well matched.

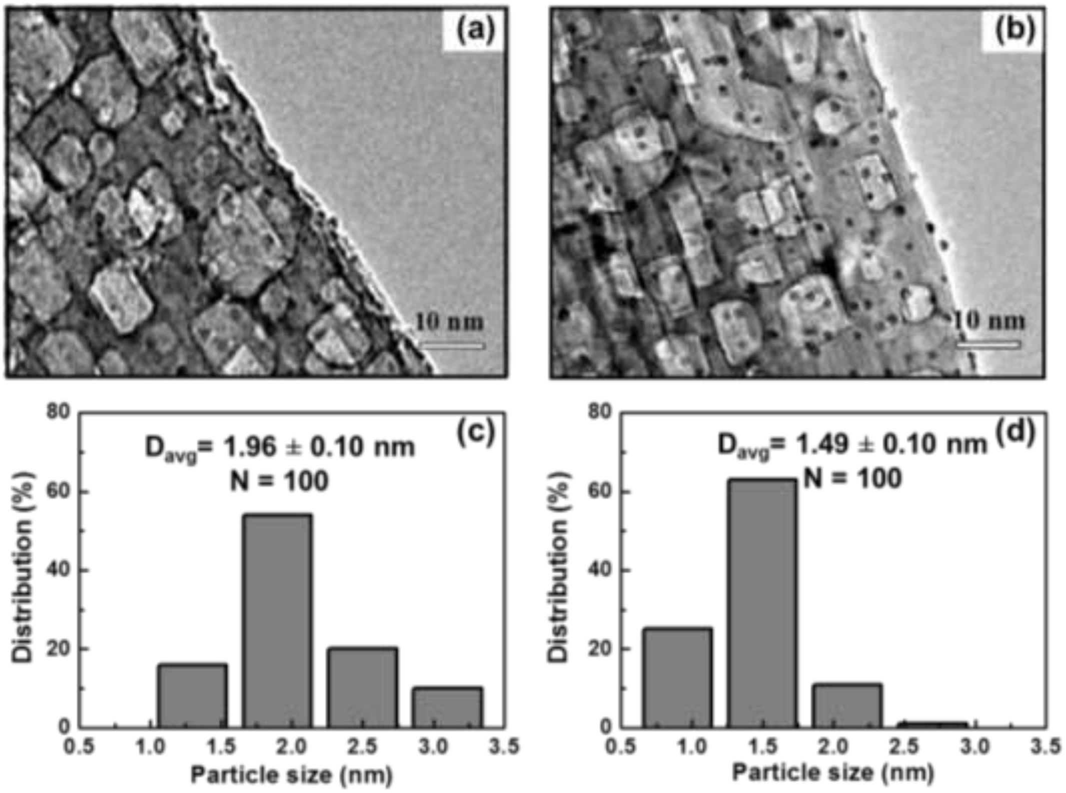

Figure S3. Typical TEM images of (a) Cu/TiO_2-NB, (b) Pt/TiO_2-NB and the corresponding cluster size distributions of (c) Cu/TiO_2-NB, (d) Pt/TiO_2-NB. The Cu nanoparticles exhibit an average diameter of 1.96 nm, and that is 1.49 nm for Pt nanoparticles. The average sizes of nanoparticles were obtained via measuring diameters of one hundred nanoparticles in TEM images.

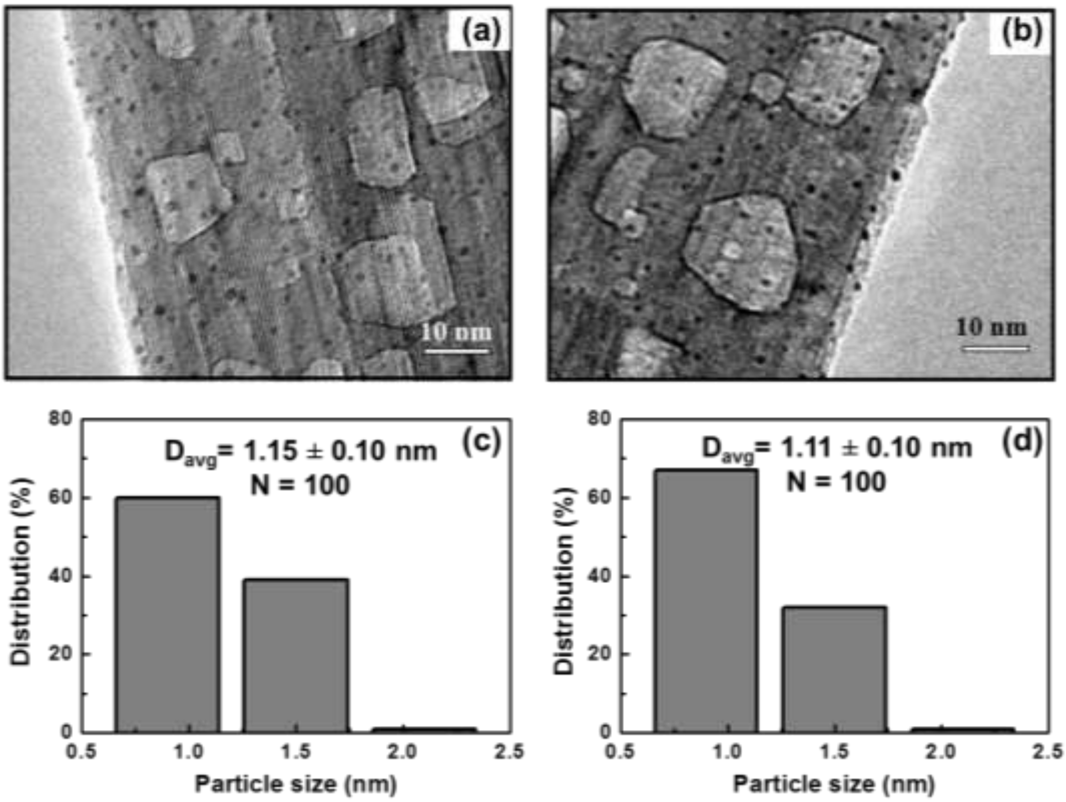

Figure S4. Typical TEM images of (a) Pt_1Cu_2/TiO_2-NB, (b) Pt_2Cu_1/TiO_2-NB and the corresponding cluster size distributions of (c) Pt_1Cu_2/TiO_2-NB, (d) Pt_2Cu_1/TiO_2-NB. The Pt_1Cu_2 nanoparticles exhibit an average diameter of 1.15 nm, and that is 1.11 nm for Pt_2Cu_1 nanoparticles. The average sizes of nanoparticles were obtained via measuring the diameters of one hundred nanoparticles in TEM images.

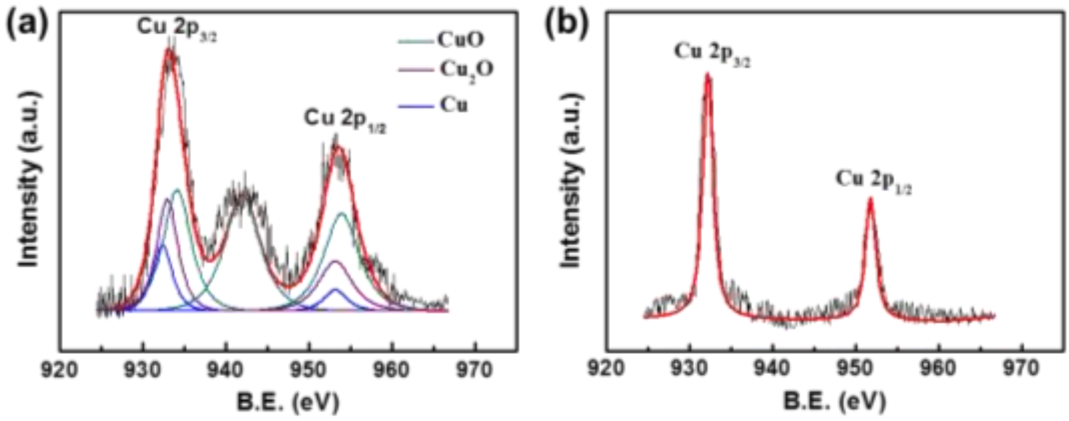

Figure S5. XPS spectra of Cu 2p in (a) Cu/TiO_2-NB and (b) Pt_1Cu_1/TiO_2-NB. As shown in Figure S5b the binding energies of Cu 2p (932.1 and 951.9 eV) in Pt_1Cu_1/TiO_2-NB can be assigned to metallic Cu, and the feature peak corresponding to CuO_x was not detected. These indicate that the oxidation of Cu atoms is effectively suppressed in bimetallic Pt-Cu alloy clusters. While, for pure Cu clusters supported on TiO_2 NBs (Figure S5a), the Cu $2p_{3/2}$ peak can be deconvolved into three Cu species Cu^0 (932.4 eV), Cu^+ (932.8 eV) and Cu^{2+} (933.9 eV),[12] indicating a CuO_x/Cu shell/core structure. This is in good agreement with the fact that the surface oxidation can occur quickly for small-sized Cu nanostructures under ambient or aqueous conditions.[13]

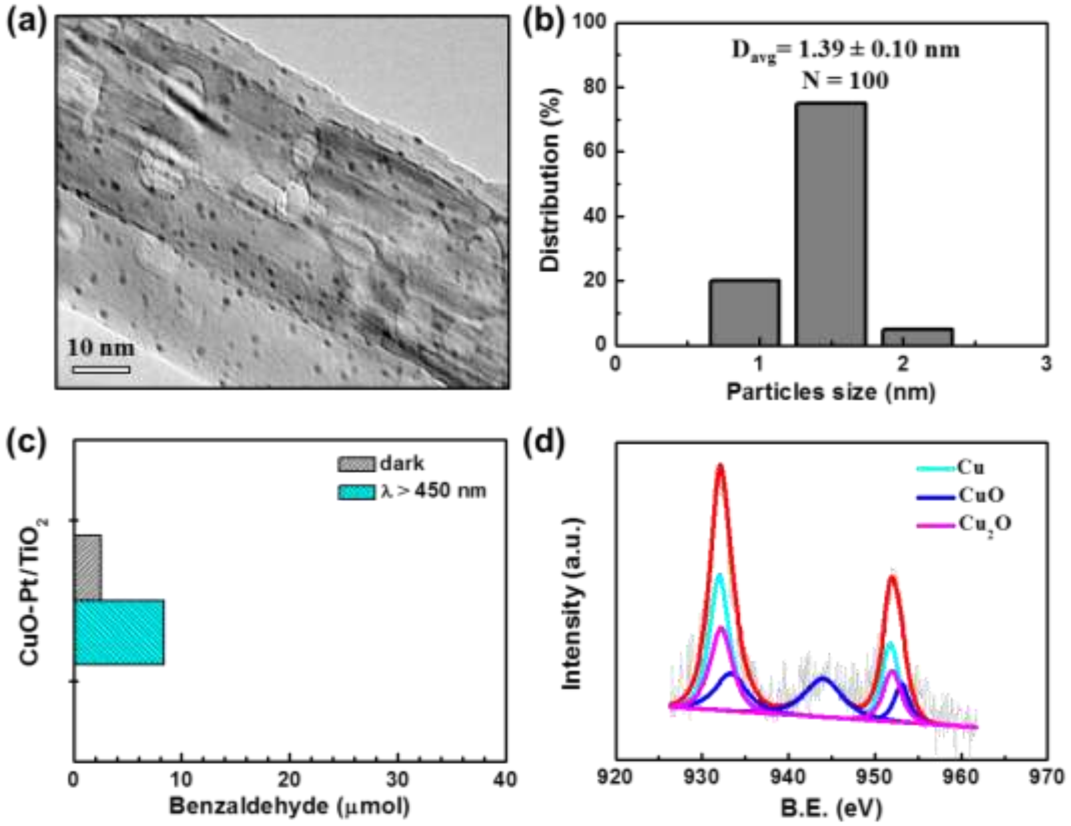

Figure S6. (a) Typical TEM image, (b) cluster size distribution, (c) amounts of benzaldehyde formed during oxidation of benzyl alcohol in the dark (shadow) and under light irradiation (white) and (d) XPS spectra of Pt-CuO_x/TiO_2-NB.

To further elucidate the alloying effect in PtCu/TiO_2-NB bimetallic catalysts, a Pt-CuO_x/TiO_2-NB nanostructure was prepared by calcining the as-deposited Pt_1Cu_1/TiO_2-NB sample in oxygen atmosphere and tested for benzyl alcohol aerobic oxidation (Figure S6). The XPS analysis confirms the formation of CuO_x species on metal clusters upon oxidation treatment which simultaneously results in the average clusters size increasing to about 1.39 nm. The amount of benzaldehyde produced over this Pt-CuO_x/TiO_2-NB catalyst in the dark is 3.4 µmol, which is much less than that obtained over the alloyed Pt_1Cu_1/TiO_2-NB catalyst (15.4 µmol), and even less than that obtained with monometallic Pt/TiO_2-NB catalyst (4.1 µmol). This result indicates that the alloying structure of Pt-Cu cluster is the key to achieve the synergistic effect for thermally catalyzing aerobic oxidation of benzyl alcohol in dark. In addition, similar reduced benzaldehyde production is also taken place for the catalytic reactions under visible light irradiation.

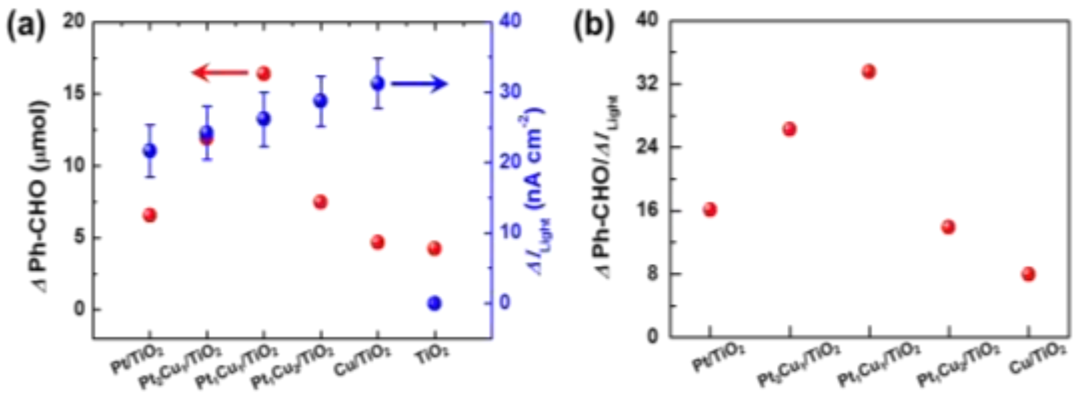

Figure S7. a) Compared with benzyl alcohol oxidation in dark, increased amount of benzaldehyde generation (*Δ*Ph-CHO) for visible light assisted catalysis on different nanostructures, and corresponding photocurrent response (ΔI_{light}) of these nanomaterials to same visible light irradiation. b) The proportion between the number of generated product molecules and the number of photo-generated electrons.

A volcano-type relationship between light-driven activity enhancement and Pt/Cu ratio was also observed (red of Figure S7a), and Pt_1Cu_1/TiO_2-NB showed the maximum light-driven activity enhancement of 16.4 μmol, which is 6.6 μmol for Pt/TiO_2-NB.

As the generated products are obtained on 20 mg nanomaterials for 5 hours, the number of reacted molecules on per unit mass (mg) nanomaterials per second were calculated as follows: (a×10^{-6} mol)×(6.023×10^{23} mol^{-1})/(5×60×60 s)/(20 mg) = a×1.67×10^{12} (mg^{-1}·s^{-1}), where a (μmol) is the quantity of products. As the photocurrents are obtained from 0.4 mg nanomaterials on 2.0 cm^{-2} electrode, the number of photogenerated electrons on per unit mass (mg) nanomaterials per second is as follows: (b×10^{-9} A cm^{-2})×(2.0 cm^{2})×(1 s)/(1.6×10^{-19} C)/(0.4 mg) = b×3.13×10^{10} (mg^{-1}·s^{-1}), where b (nA cm^{-2}) is the magnitude of photocurrent. With above conversion coefficients, the increased number of generated benzaldehyde molecules on per unit mass (mg) nanomaterials per second for visible light assisted catalysis on different nanostructures and corresponding number of photogenerated electrons on per unit mass (mg) nanomaterials per second can be calculated out, which were shown in Figure 2f of the main text.

In Figure S7b, a volcano-type relationship between generated molecules/electrons ratios and Pt/Cu ratios was also observed, and a maximum ratio of 33.5 was obtained for Pt_1Cu_1/TiO_2-NB. Considering that the metal clusters have similar light absorption during the two measurements and the photo-generated hot electrons are effectively collected for producing photocurrents, it can be reckoned that one photo-generated hot hole in Pt_1Cu_1 clusters can bring out the generation of 33.5 additional reacting molecules.

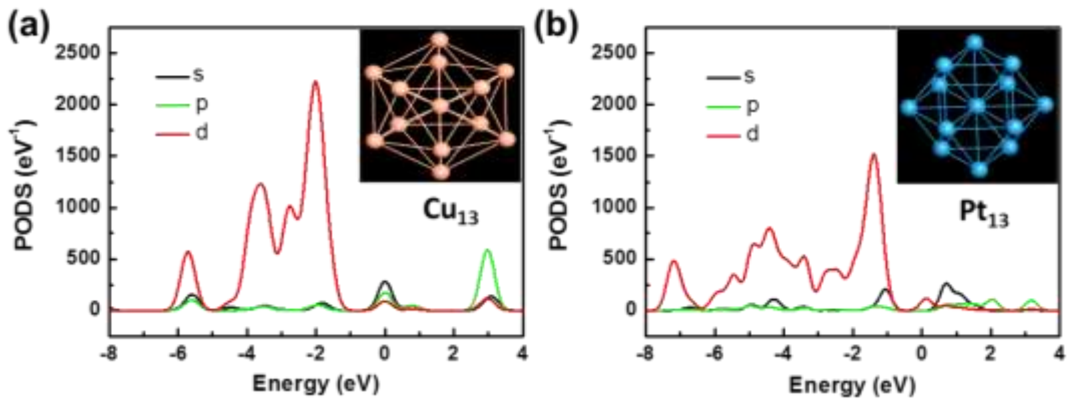

Figure S8. a) Projected density of states on orbitals (s, p, and d) for model Cu_{13} (a) and Pt_{13} (b) clusters. Insets are the optimized Cu_{13} and Pt_{13} structures for calculations.

To further compare the interband excitation for Cu and Pt atoms, projected density of states (PDOS) of orbitals for model Cu_{13} and Pt_{13} clusters were studied by theoretical calculation (Figure S8). As shown in Figure 2d and Figure S8, near Fermi level the PDOS of d orbits for Cu is larger than that for Pt, thus generating more hot carriers by photoexciting from d to sp orbitals for Cu atoms, which is consistent with the results of optical absorption (Figure 2c). Therefore, in Pt-Cu clusters, most hot carriers are generated by interband excitation of Cu atoms from d to sp orbitals.

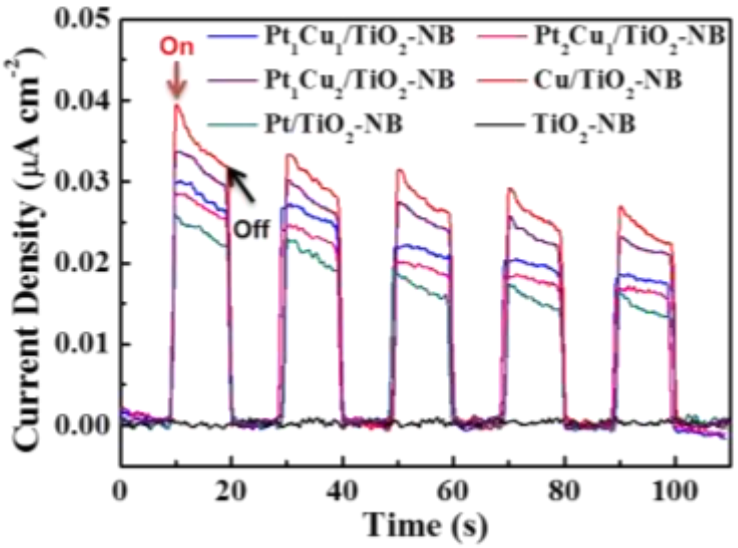

Figure S9. The photocurrent responses of TiO_2-NB, Cu/TiO_2-NB, Pt/TiO_2-NB, Pt_2Cu_1/TiO_2-NB, Pt_1Cu_1/TiO_2-NB and Pt_1Cu_2/TiO_2-NB nanomaterials to visible light on and off.

With increasing the Cu/Pt ratio enhanced photocurrent can be observed (Figure S9). The biggest photocurrent response was obtained for Cu/TiO_2-NB, which is consistent with its strong light absorption characteristics (Figure 2c). This suggests that a large amount of energetic electrons would be generated in Cu_xO/Cu clusters upon visible light irradiation and then injected into TiO_2. However, the numerous photoexcited electrons in Cu/TiO_2-NB did not contribute to any activity enhancement compared to bare TiO_2 nanobelts under visible light irradiation (Figure 2a). This result suggests that the light-promoted aerobic oxidation of benzyl alcohol on PtCu/TiO_2-NB catalysts is a metal cluster dominated photocatalytic process, and CuO_x is inactive for this reaction.

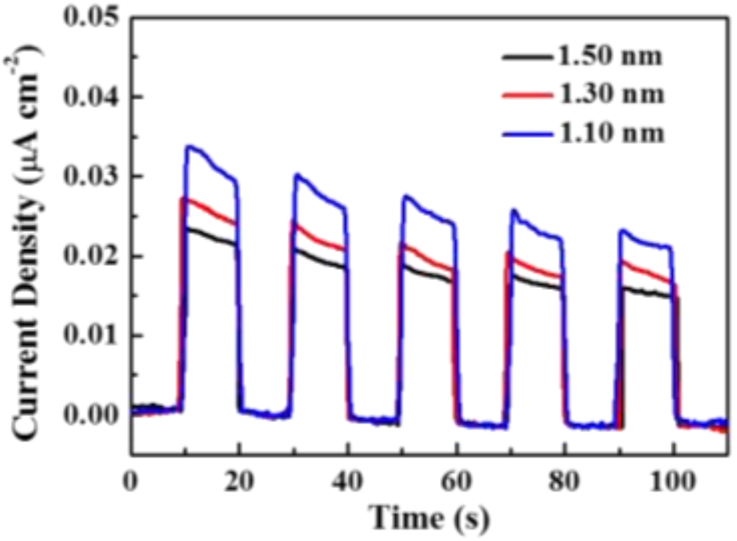

Figure S10. The photocurrent responses of Pt_1Cu_1/TiO_2-NB nanomaterials with different cluster sizes of 1.1, 1.3 and 1.5 nm to visible light on and off.

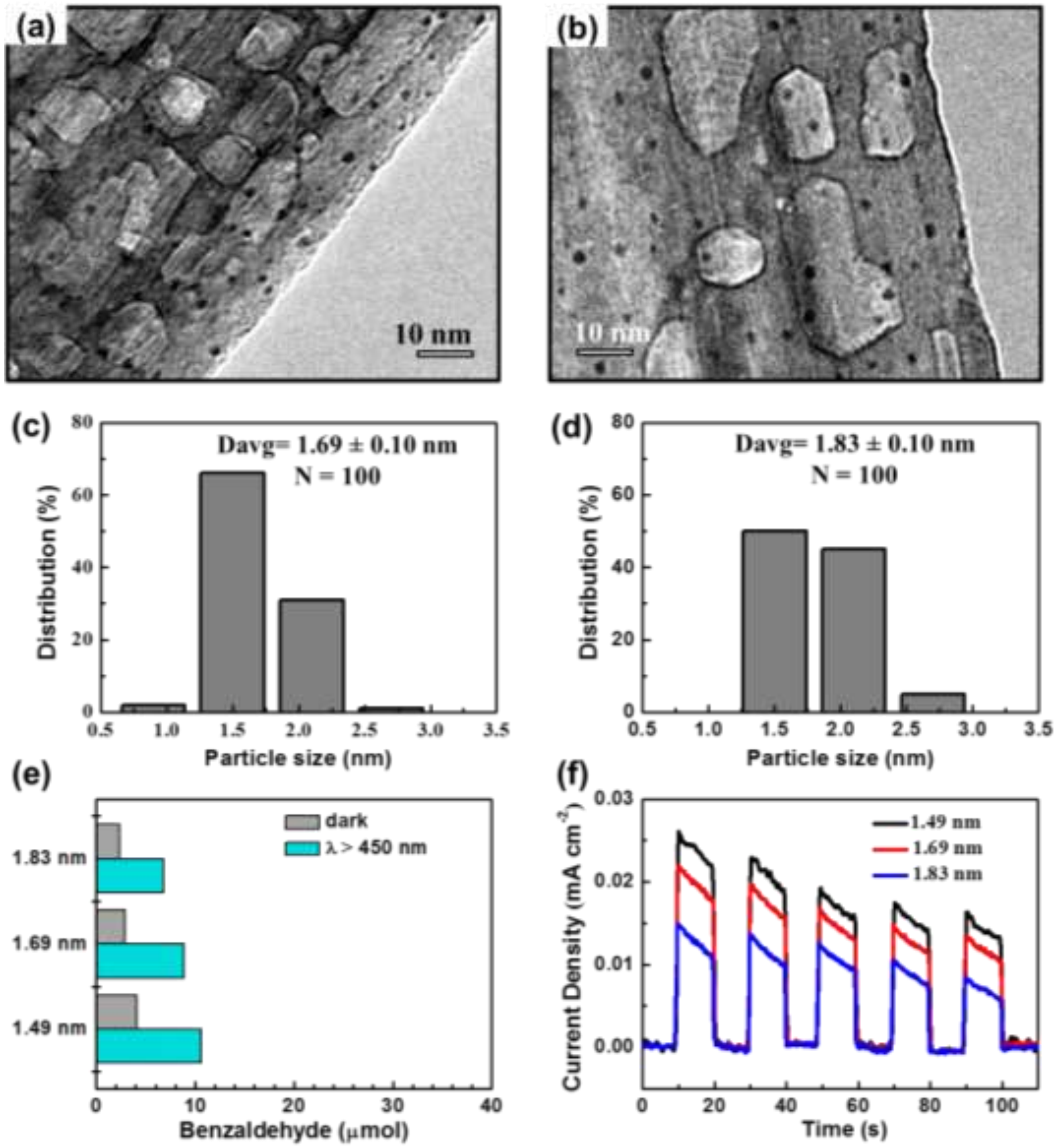

Figure S11. Typical TEM images of Pt/TiO_2-NB pretreated under H_2 flow for (a) 4 h, (b) 6 h and the corresponding cluster size distributions (c) 4 h, (d) 6 h. (e) Amounts of benzaldehyde formed during oxidation of benzyl alcohol in the dark (shadow) and with visible light irradiation (white) as well as (f) the photocurrent responses of Pt/TiO_2-NB nanostructures pretreated under H_2 flow for 2 h (1.49 nm), 4 h (1.69 nm), 6 h (1.83 nm) to visible light on and off.

3. Table S1–S4

Table S1. The composition and atom ratios of $PtCu/TiO_2$-NB. (The results were detected by ICP analysis.)

Catalysts	Cu/wt.%	Pt/wt.%	(Cu+Pt)/wt.%	Pt/Cu atomic ratio
Cu/TiO_2-NB	0.564	0	0.564	0:1
Pt_1Cu_2/TiO_2-NB	0.191	0.342	0.533	0.59:1
Pt_1Cu_1/TiO_2-NB	0.119	0.410	0.529	1.13:1
Pt_2Cu_1/TiO_2-NB	0.067	0.441	0.508	2.17:1
Pt_4Cu_1/TiO_2-NB	0.037	0.484	0.521	4.33:1
Pt/TiO_2-NB	0	0.507	0.507	1:0

Table S2. Amounts of benzaldehyde formed during oxidation of benzyl alcohol in the dark and with light irradiation as well as light-driven increment for as-prepared catalysts.

Catalysts	Dark/μmol	Light/μmol	The increment/μmol
Pt_1Cu_2/TiO_2-NB	14.31	21.76	7.45
Pt_2Cu_1/TiO_2-NB	10.70	22.59	11.89
Pt_1Cu_1/TiO_2-NB (1.1 nm)	15.40	31.81	16.41
Pt_1Cu_1/TiO_2-NB (1.3 nm)	10.30	20.44	10.14
Pt_1Cu_1/TiO_2-NB (1.5 nm)	9.41	16.14	6.73
Pt/TiO_2-NB (2 h)	4.10	10.66	6.56
Pt/TiO_2-NB (4 h)	2.93	8.91	5.98
Pt/TiO_2-NB (6 h)	2.35	6.86	4.51
$Pt-CuO_x/TiO_2$-NB	3.41	8.39	4.98

Table S3. Calculated work function for Pt and Pt-Cu alloy clusters supported on TiO_2-NB.

Samples	Diameter/nm	Work function/eV
Pt/TiO_2-NB	1.49	6.37
	1.69	6.29
	1.83	6.24
Pt_1Cu_2/TiO_2-NB	1.15	5.92
Pt_2Cu_1/TiO_2-NB	1.11	6.29
Pt_1Cu_1/TiO_2-NB	1.10	6.13
	1.30	5.98
	1.50	5.87

Through considering both work function of intrinsic metal and small size effect of nano-clusters, the work functions for origin Pt-Cu clusters were calculated by using the equations given below: [14]

$$W_m = W_{m,\infty} + 1.08/d \quad (1)$$

and

$$W_{alloy,\infty} = xW_{Pt,\infty} + (1-x)\, W_{Cu,\infty} \quad (2)$$

$W_{m,\infty}$ and W_m are the work functions of bulk metal and corresponding metal clusters and x means the atomic percentage of Pt. The $W_{Pt,\infty}$ and $W_{Cu,\infty}$ are 5.65 and 4.65 eV, respectively. [15]

With increasing the work functions for clusters with smaller size, higher Schottky barriers are expected to form at cluster/TiO_2-NB interfaces, which could suppress the hot electrons in the conduction band of TiO_2 to recombine with the stayed hot holes in the clusters, [16] thus generating long lifetime hot holes in the clusters for further catalytic reactions.

Table S4. Hot electron-hole pair lifetimes in Pt_1Cu_1/TiO_2-NB (1.1 nm) nanostructures

	1	2	3	4	5
***τ* (s)**	24.44	23.83	22.18	23.11	22.45
χ^2	0.9882	0.9829	0.9878	0.9931	0.9941

The average hot electron-hole pair lifetimes in Pt_1Cu_1/TiO_2-NB (1.1 nm) nanostructures were obtained from the single exponential fitting the transient open-circuit voltage (V_{oc}) decay during termination of irradiation, which was shown out in Figure 2e. τ means the rate parameter of the decay process. χ^2 means the r-square value of the fitted curves. Therefore, the average lifetime of hot electron-hot pairs is 23.21 ± 0.84 s.

References

[1] Z. Yuan, B. Su, *Colloids Surf. A*, **2004**, *241*, 173.

[2] X. Chen, S. Mao, *Chem. Rev.* **2007**, *107*, 2891.

[3] E. Apra, A. Fortunelli, *J. Mol. Struct. (Theochem)*. **2000**, *501-502*, 251.

[4] N. Watari, S. Ohnishi, *Phys. Rev. B.* **1998**, *58*, 1665.

[5] K. Baishya, J. C. Idrobo, S. Öğüt, M. Yang, K. A. Jackson, J. Jellinek, *J. Phys. Rev. B.* **2011**, *83*, 245402.

[6] M. Yang, K. A. Jackson, C. Koehler, T. Frauenheim, J. Jellinek, *J. Chem. Phys.* **2006**, *124*, 024308.

[7] B. *J.* Delley, *Chem. Phys*. **1990**, *92*, 508.

[8] B. *J.* Delley, *Chem. Phys*. **2000**, *113*, 7756.

[9] Z. J. Yang, H. B. Ji, *ACS Sustainable Chem. Eng.* **2013**, *1*, 1172.

[10] Z. J. Yang, H. G. Jiang, X. T. Zhou, Y. X. Fang, H. B. Ji, *Supramol. Chem.* **2012**, *24*, 379.

[11] B. Liu, A. Khare, E. S. Aydil, *ACS Appl. Mater. Interfaces* **2011**, *3,* 4444.

[12] J. F. Moulder, W. F. Stickle, P. E. Sobol, K. D. Bomben, *Handbook of X-ray Photoelectron Spectroscopy, Physical Electronics,* Inc., Eden Prairie, Minnesota, **1995**.

[13] Q. Jia, D. Zhao, B. Tang, N. Zhao, X. Xu, H. Liu, *J. Mater. Chem. A* **2014**, *2*, 16292.

[14] D. M. Wood, *Phys. Rev. Lett.* **1981**, *46*, 749.

[15] D. E. Eastman, *Phys. Rev. B* **1970**, *2*, 1.

[16] Y. Shiraishi, D. Tsukamoto, Y. Sugano, A. Shiro, S. Ichikawa, S. Tanaka, T. Hirai, *ACS Catal*. **2012**, *2*, 1984.
